# Metformin decreases bacterial trimethylamine production and trimethylamine N-oxide levels in db/db mice

**DOI:** 10.1038/s41598-020-71470-4

**Published:** 2020-09-03

**Authors:** Janis Kuka, Melita Videja, Marina Makrecka-Kuka, Janis Liepins, Solveiga Grinberga, Eduards Sevostjanovs, Karlis Vilks, Edgars Liepinsh, Maija Dambrova

**Affiliations:** 1 Latvian Institute of Organic Synthesis, Aizkraukles Str. 21, Riga, 1006 Latvia; 2 Faculty of Pharmacy, Rīga Stradiņš University, Dzirciema Str. 16, Riga, 1007 Latvia; 3 Institute of Microbiology and Biotechnology, University of Latvia, Jelgavas Str. 1, Riga, 1004 Latvia

**Keywords:** Biomarkers, Endocrinology

## Abstract

The current study aimed to explore whether metformin, the most widely prescribed oral medication for the treatment of type 2 diabetes, alters plasma levels of cardiometabolic disease-related metabolite trimethylamine N-oxide (TMAO) in db/db mice with type 2 diabetes. TMAO plasma concentration was up to 13.2-fold higher in db/db mice when compared to control mice, while in db/db mice fed choline-enriched diet, that mimics meat and dairy product intake, TMAO plasma level was increased 16.8-times. Metformin (250 mg/kg/day) significantly decreased TMAO concentration by up to twofold in both standard and choline-supplemented diet-fed db/db mice plasma. In vitro, metformin significantly decreased the bacterial production rate of trimethylamine (TMA), the precursor of TMAO, from choline up to 3.25-fold in *K. pneumoniae* and up to 26-fold in *P. Mirabilis*, while significantly slowing the growth of *P. Mirabilis* only. Metformin did not affect the expression of genes encoding subunits of bacterial choline-TMA-lyase microcompartment, the activity of the enzyme itself and choline uptake, suggesting that more complex regulation beyond the choline-TMA-lyase is present. To conclude, the TMAO decreasing effect of metformin could be an additional mechanism behind the clinically observed cardiovascular benefits of the drug.

## Introduction

Type 2 diabetes is characterized by alterations in the composition of intestinal microbiota, notably by a decrease in butyrate-producing bacteria and an increase in opportunistic pathogens, bacteria that can exploit weakened immune system to cause an infection^1,2^. While gut bacteria have been known to play fundamental roles in the pathogenesis of many diseases, such as obesity, diabetes and cardiovascular disease, a clear link underlying these roles was missing. A breakthrough in predicting cardiometabolic risks and potential outcomes based on intestinal microbiota composition came when trimethylamine N-oxide (TMAO) was linked to the development and progression of cardiovascular diseases^3,4^. Thus, it was shown that intestinal microbiota produces trimethylamine (TMA) from dietary tertiary amines like choline, carnitine and butyrobetaine. Upon entering circulation TMA is further metabolized to TMAO by host liver enzyme flavin-containing monooxygenase 3 (FMO3). Recently it was shown that type 2 diabetes is also associated with higher plasma level of TMAO^5,6^.

In recent years, an interesting relationship between TMAO, insulin and insulin resistance-induced cardiovascular diseases has been elucidated. Thus, it was shown that the expression of TMAO producing enzyme FMO3 is suppressed by insulin^7^; subsequently, it was shown that FMO3 knockout mice are protected from the development of hyperglycaemia, hyperlipidaemia and atherosclerosis due to the suppression of FOXO1, a transcription factor that is the main target of insulin signalling. Very recently, it was shown that the endoplasmic reticulum stress kinase PERK acts as a receptor for TMAO; thus, TMAO at physiologically relevant concentrations selectively activates PERK, which in turn induces the transcription factor FOXO1^8^ and promotes metabolic dysfunction.

The most widely prescribed medication for the treatment of type 2 diabetes is metformin. However, approximately one fourth of patients receiving metformin report side effects directly attributed to the therapy, and almost 62% of side effects reported are gastrointestinal ones such as nausea and diarrhoea^9^ suggesting of changes in microbiota composition. Indeed, recent findings indicate that both therapeutic effects and side effects of metformin at least to some extent depend on drug use-induced changes in intestinal microbiota^10,11^. It has been shown that metformin treatment can partially normalize the aberrant Firmicutes/Bacteroidetes ratio in high-fat diet-fed mice by decreasing *Firmicutes* and restoring *Bacteroidetes* and *Verrucomicrobia* levels^12^ and improve the function of a high-fat diet impaired glucose-SGLT1-sensing glucoregulatory pathway in rats^13^. Metformin-induced intestinal effects might be related to relatively high mM concentrations than can be achieved in the lumen of intestine following oral administration of the drug at higher doses^14^. Although anti-diabetic effects and more recently^15,16^ beneficial cardiovascular effects in diabetes and non-diabetes patients of metformin are well characterized, molecular mechanisms and particularly biochemical mechanisms involving the effects on intestinal microbiota remain to be elucidated. First, the experimental setup of the present study aimed to explore the effects of metformin on TMAO levels under conditions of type 2 diabetes in db/db mice. Second, since TMA and subsequently TMAO are mostly formed from dietary tertiary amines, mainly from choline and carnitine/butyrobetaine^4,17,18^, additional experiments were performed to evaluate the efficacy of metformin in db/db mice under increased choline load to mimic high meat and dairy product (rich in choline, carnitine and other tertiary amines/their derivatives) intake as in typical Western diet. Third, the direct effects of metformin on opportunistic pathogens present in human intestines that differ significantly in their ability to produce TMA from different sources were evaluated in vitro. Thus, *Klebsiella pneumoniae* was chosen because it can produce TMA from all the main precursors—choline (via *CutC/D* (choline-TMA-lyase complex)) and carnitine/butyrobetaine (via *CntA/B* (Carnitine monooxygenase complex)), while *Proteus mirabilis* was chosen because it has just *CutC/D* and can produce TMA only from choline^19,20^.

## Results

### Effects on TMAO production in db/db mice fed standard chow and diet supplemented with choline

**Figure 1:**
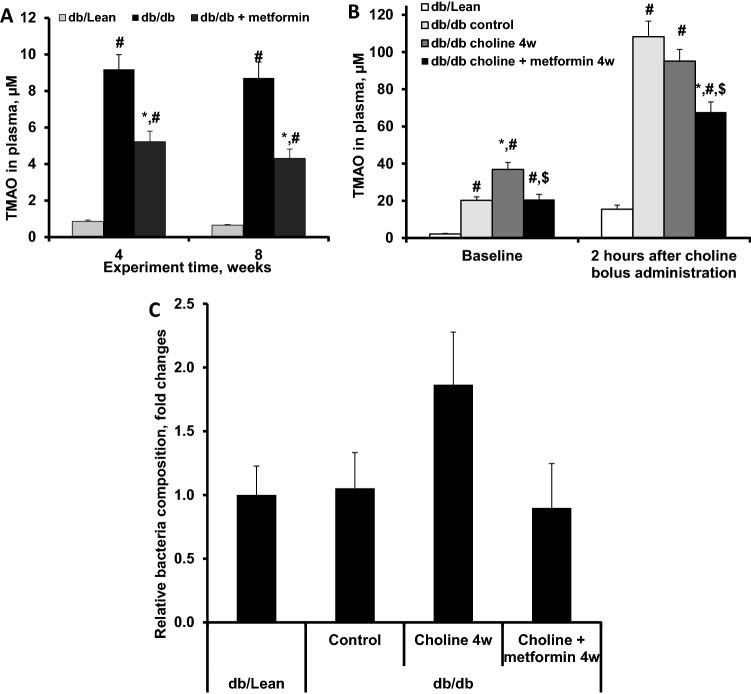
Effects of metformin (250 mg/kg) treatment on TMAO plasma concentrations and relative bacteria composition in db/db mice. Panel A, effects of 4 and 8 weeks of treatment with metformin in mice fed a standard laboratory diet; Panel B, effects of 4 weeks of metformin treatment in mice supplemented with choline. Panel C, effects of 4 weeks of metformin treatment in mice supplemented with choline on relative presence of *P. mirabilis* in gut microbiota. The results are the mean of 8 animals ± SEM in the db/db and db/db + metformin groups and 10 animals in the db/Lean group for panel A. The results are the mean of 10 animals ± SEM for panel B. The results are mean of 9–10 animals for panel C, one sample was excluded in choline group and one in choline + metformin group after identified as outliers by ROUT analysis. * Significantly different from the respective db/db control group, ^#^ significantly different from the respective db/Lean group, ^$^ significantly different from the respective db/db + choline 4w group (ANOVA followed by Tukey’s test; *P* < 0.05).

The TMAO concentration in plasma of db/db control mice fed standard chow (R70) was significantly, up to 13.2-fold, higher than that in db/Lean mice plasma. Metformin administration to db/db mice at a dose of 250 mg/kg for 8 weeks significantly decreased TMAO levels up to 2.0-fold when compared to those of db/db control mice (Fig. 1A).

Because TMA and subsequent TMAO production depend on diet, we next evaluated whether metformin treatment can affect chronic and acute dietary choline load-induced increases in TMAO levels. Based on findings with standard chow, where the efficacy of metformin was similar after 4 and 8 weeks of treatment, administration of metformin for 4 weeks was chosen for follow-up studies using a choline-supplemented diet. In this experiment, choline pretreatment for 4 weeks resulted in a further 1.8-fold increase in basal TMAO levels (36.9 µM) when compared to db/db mice on a standard diet (Fig. 1B) and 16.8-fold increase when compared to control db/Lean mice. Two h after acute choline load, TMAO plasma levels increased in all experimental groups, and highest concentrations up to 95–108 µM were observed in the db/db control and choline pre-treated groups. In db/db mice on a diet supplemented with choline, metformin treatment significantly decreased both basal and 2 h post-choline-load TMAO levels (Fig. 1B). The choline plasma concentration in db/db control mice plasma was 45 µM before and 58 µM after acute choline load, and no significant changes in choline plasma concentrations due to treatment with metformin were observed among experimental groups.

To assess the population of selected bacteria in the faecal samples (from mice treated with choline or choline in combination with metformin for 4 weeks) we evaluated metformin-induced changes in abundance of *P. mirabilis* and *K. pneumoniae.* While levels of *K. pneumoniae* were too low to be detected, *P. mirabilis* bacteria were detected and we observed tendency for relatively higher *P. mirabilis* bacteria presence in db/db mice that received choline and also tendency for metformin to prevent this increase (Fig. 1C). In addition, we investigated the effect of metformin on bacterial genera *Olsenella* and *Desulfovibrio,* members of which are known to be able to produce TMA from choline^21^. Metformin treatment resulted in a significant relative population increase of *Desulfovibrio* species (Supplementary Fig. 1). Flavin-containing monooxygenase 3 (FMO3) activity in the liver microsomal fraction in the presence of metformin was measured and metformin did not change FMO3 activity at concentrations up to 2 mM (Supplementary Fig. 2).

**Figure 2:**
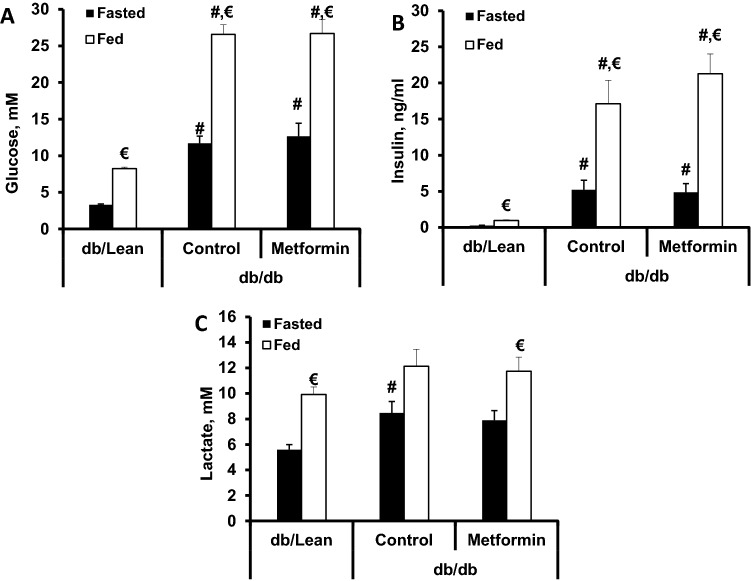
Effects of an 8-week metformin treatment at a dose of 250 mg/kg on blood glucose levels in fasted and fed states (**A**), plasma insulin in fasted and fed states (**B**) and plasma lactate levels in fasted and fed states (**C**). The results are the mean ± SEM of 8 animals in the db/db and db/db + metformin groups and 10 animals in the db/Lean mice group. ^#^*P* < 0.05 vs. db/Lean group (ANOVA, followed by Tukey’s test for A, Kruskal–Wallis test followed by Dunn’s multiple comparison test for B and C). ^€^ significantly different from the respective group at fasted state (paired t-test).

Glucose and insulin plasma concentrations were significantly increased in both fed and fasted states, and metformin had no effect in any of these states (Fig. 2A,B). Lactate plasma concentration was significantly increased only in the fasted state in db/db control mice plasma but not in metformin-treated db/db mice plasma (Fig. 2C). Taken together, metformin treatment had no glucose-lowering and insulin sensitivity-improving effect in db/db mice with type 2 diabetes; thus, the effects observed in this study were independent of glucose and insulin plasma concentrations.

### Metformin effects on bacterial TMA production

**Figure 3:**
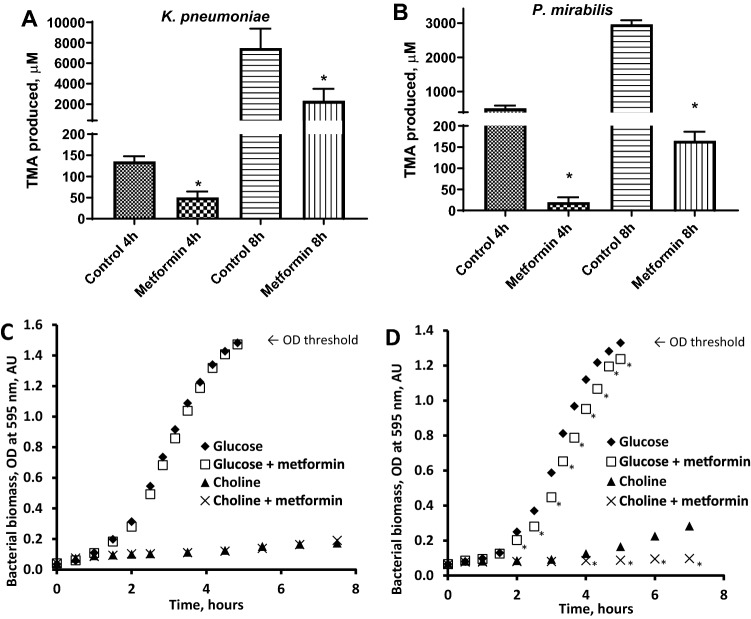
Metformin (27 mM) effects on TMA production from choline (**A**, **B**) and bacterial growth (**C**, **D**) in *K. pneumoniae* (**A** and **C**) and *P. mirabilis* (**B** and **D**) under anaerobic conditions. The results are the mean ± SD of 3 independent replicates for A and 4 for B, C and D. * *P* < 0.05 vs. respective time point control (t-test). The maximum absorbance value for turbid suspensions in the colorimeter used in the experiments was 1.4.

Since treatment with metformin significantly decreased TMAO production in mice, we tested whether this effect could be related to changes in ability of gastrointestinal tract bacteria to produce TMA, which is required for host liver to produce TMAO. For this we chose human gastrointestinal tract bacteria *K. pneumoniae* and *P. mirabilis* both known to be able to produce TMA^19,20^. Metformin significantly decreased TMA production 3.25-fold in *K. pneumoniae* (Fig. 3A), while bacterial growth of *K. pneumoniae* was not affected (Fig. 3C) when choline or glucose was used as the sole carbon source for up to 8 h. Metformin significantly decreased TMA production (up to 26-fold) in *P. mirabilis* (Fig. 3B). Unlike with *K. pneumoniae*, metformin significantly decreased *P. mirabilis* bacterial biomass growth when choline was used as the sole carbon source. The effect was present, albeit less pronounced when *P. mirabilis* was grown in media with glucose as the sole carbon source (Fig. 3D). Decrease in choline concentration in incubation media indicated that choline is converted to TMA by bacteria (Supplementary Fig. 3).

**Figure 4:**
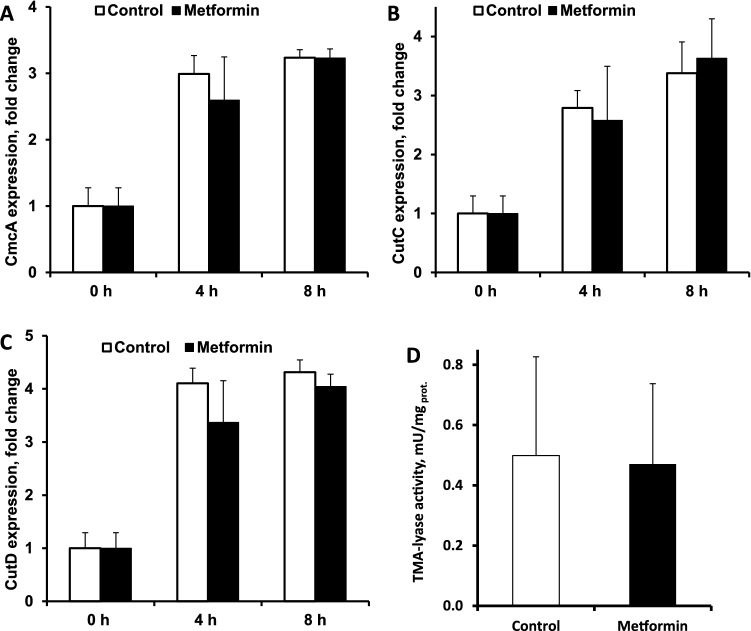
Effects of metformin (27 mM) under anaerobic conditions on the expression of genes that encode enclosure microcompartment structural proteins (**A**) and choline-TMA-lyase subunit proteins (**B** and **C**) in *K. pneumoniae* and on the choline-TMA-lyase activity in *P. mirabilis* bacterial lysate (**D**). The results are the mean ± SD of 3 independent replicates for A, B and C and the mean of 5 independent replicates for D.

To determine if the administration of metformin changes the expression of bacterial genes encoding key proteins of choline metabolism, choline lyase enzyme complex subunits cutC and cutD and enzyme enclosure microcompartment structural protein cmcA^22,23^, *K. pneumoniae* were cultivated with choline (0.5% in broth) as the sole carbon source, and metformin was added at the final concentration of 27 mM (0.5% in broth). Transcription samples were extracted after 0, 4 and 8 h of anaerobic cultivation with choline and with or without metformin. The expression of the choline-TMA-lyase complex subunits (cutC and cutD) and the microcompartment structural gene (cmcA) increased over time; however, metformin had no significant effect on the expression of any of the tested genes (Fig. 4A,B,C). Moreover, choline-TMA-lyase activity was determined in *P. mirabilis* bacterial lysate, and we found no metformin effect on the TMA production rate (Fig. 4D).

Because metformin did not affect the expression of genes coding choline-TMA-lyase and enzymatic activity, we next evaluated its effects on choline uptake. To confirm bacterial viability and functional capacity, carnitine uptake was tested as a positive control as described previously^19^; [^3^H]-carnitine uptake was time-dependent with a maximal velocity of 0.77 ± 0.07 fmol/min/mg bacterial protein, indicating that the bacteria were viable and functional.[^3^H]-choline was used for uptake measurements in *K. pneumoniae,* and metformin was added at a final concentration of 27 mM. The choline concentration in bacteria after 10 min of incubation was 1.49 fmol/mg bacterial protein and was unchanged by metformin treatment (1.50 fmol/mg bacterial protein).

## Discussion

Knowledge on the mechanism of the anti-diabetes action of metformin has become increasingly complex over the years from complex I inhibition to suppression of gluconeogenesis by inhibition of mitochondrial glycerophosphate dehydrogenase^24^ to interaction with intestinal microbiota^10,25^ to the suggested potential iron chelating activity of metformin^26^. We now show that metformin is able to directly inhibit bacterial TMA production and decrease TMAO availability in mice. In this study db/db mice had pronounced hyperglycaemia and obesity; under our setup, treatment with metformin did not affect blood glucose and insulin levels, indicating that metformin-induced changes in TMAO levels are at least partially independent of glucose and insulin plasma concentrations.

TMAO has recently been shown to cause endothelial dysfunction through cellular inflammation, elevation of oxidative stress and suppression of endothelial progenitor cell functions^27–29^. In turn, in the clinical setting, metformin treatment is beneficial in attenuating endothelial dysfunction in patients with prediabetes, metabolic syndrome and type 1 and 2 diabetes^30,31^. While decreasing glucose levels and improving insulin sensitivity are largely responsible for the beneficial effects of metformin, in light of the present findings, there is a basis for attributing part of the protective effects of metformin on the cardiovascular system to the decrease in circulating pro-atherogenic TMAO levels. Indeed, in recent years, metformin treatment has been shown to improve the endothelial glycocalyx barrier in db/db mice without changing blood glucose levels^32^ and to improve endothelial function in spontaneously hypertensive rats with type 1 diabetes independent of glycaemia control^33^.

Previously, we have shown that 12-week-old db/db mice have a significantly elevated TMAO level of 9 µM^5^, which, if translated to human patients, indicates a significantly increased risk of major adverse cardiovascular events as the TMAO plasma level is above the risk threshold of 6.18 µM^3^. It has been recently reported that TMAO levels in db/db mice are higher during the dark cycle^34^, and plasma samples in the current study were also collected during the dark cycle. Thus, in db/db control mice fed a standard laboratory R70 diet, TMAO plasma levels were well above 6.18 µM, while treatment with metformin decreased TMAO levels approximately two-fold and, importantly, below the threshold of TMAO plasma levels associated with increased risk of major cardiovascular complications. In animals on a diet supplemented with choline metformin treatment significantly decreased TMAO plasma concentration almost 1.8-times; however, TMAO plasma levels remained higher than the risk threshold. It should be noted that our experimental results differ from the previously published effects of metformin treatment on TMAO levels in patients. Thus, in metabonomics study metformin administration (dose not stated) was associated with increased TMAO availability^35^, while interventional cross-over study where metformin was used at a dose of 2 g per day^36^ showed no effect on TMAO levels. However, in both studies there were no indications that the patients were controlled for seafood intake. Seafood is very rich in TMAO and consumption of fish or fish products should be clear exclusion criteria when looking at TMAO plasma availability^5,37^. Given our present data and previously reported metformin concentrations that can be reached in the intestines after the use of high doses^14,38^, dose of 2 g per day as in the study by Velebova and colleagues^36^ should have been sufficient to induce decrease in bacterial TMA production. It must be stressed that we did determine effects of metformin on abundance of only selected intestinal bacteria and their ability to produce TMA in mice. Further studies would be required to show how in vitro findings and data from db/db mice model translate to clinical setting under diet controlled conditions, and how metformin changes the abundance of selected bacteria that produce TMA from tertiary dietary amines in patients. Taken together, although metformin can decrease plasma TMAO availability in mice below the risk threshold, dietary changes would likely be required to reach clinically relevant endpoints and follow-up study in patient population is warranted.

Our findings strongly imply that acute effects of metformin result in overall decrease in TMAO availability through decreased bacterial TMA production. We found that metformin treatment does not result in uniform alterations in TMA-producing bacteria (both decrease and increase in bacterial population can be observed) and does not inhibit FMO3 activity. The effect of metformin on TMA synthesis also appears to be rather complex. Choline degradation to TMA in bacteria occurs in specialized microcompartments that contain choline-TMA-lyase^39^. Our data imply that other components or processes like co-factor recycling in these microcompartments but not choline-TMA-lyase itself are affected by metformin resulting in overall inhibition of TMA production. The current results prove that metformin inhibits microbial TMA production from choline; metformin is unable to decrease TMA production in *K. pneumoniae* when carnitine instead of choline is used as the sole substrate (unpublished observations). Metformin has no effect on anaerobic growth of *K. pneumoniae* (omnivorous tertiary amine metabolizer) but significantly delays (bacteriostatic effect) growth of *P. mirabilis* (choline-only metabolizing bacteria) under the same experimental conditions. To conclude, we present proof that metformin can induce significant changes in TMAO levels likely due to direct non-lethal inhibition of bacterial TMA production. Moreover, these effects are independent of glucose and insulin plasma concentrations and could be an additional mechanism behind the known cardiovascular benefits of metformin therapy.

## Materials and methods

### Animals and treatment

Sixteen male db/db (BKS.Cg- + Leprdb/ + Leprdb/OlaHsd) mice and 10 age-matched non-diabetic db/Lean (db/ + (BKS.Cg- + Leprdb/ + /OlaHsd)) male mice (10 weeks old, Envigo, Venray, Netherlands) were housed for two weeks prior to treatment under standard conditions (21–23 °C, reverse 12-h light/dark cycle, relative humidity 45–65%) with unlimited access to water and food (R70 diet from Lantmännen, (Stockholm, Sweden)). The experimental procedures involving animals were performed in accordance with the guidelines of the European Community and local laws and policies, and all of the procedures were approved by Latvian Animal Protection Ethical Committee, Food and Veterinary Service, Riga, Latvia. Studies involving animals are reported in accordance with the ARRIVE guidelines^40,41^. Db/db mice were randomly divided into two experimental groups and given daily oral doses of water (db/db control group, n = 8) or 250 mg/kg metformin (db/db metformin group, n = 8) for 8 weeks. Db/Lean mice (n = 10) were used as a control. Plasma samples were collected after 4 and 8 weeks of treatment. To test metformin (TCI Europe N.V., Zwijndrecht, Belgium) effects in case of increased tertiary amine load, choline (TCI EUROPE N.V., Zwijndrecht, Belgium) was chosen as the most common food tertiary amine.

Another 30 db/db male mice (BKS.Cg- + Leprdb/ + Leprdb/OlaHsd) and 10 age-matched non-diabetic db/Lean (db/ + (BKS.Cg- + Leprdb/ + /OlaHsd)) male mice (10 weeks old, Envigo, Venray, Netherlands) were obtained for the follow-up experiment based on data obtained from the first study. Db/db mice were divided into three experimental groups and given daily oral doses of water (db/db control group, n = 10), 0.5% choline in drinking water for 4 weeks to facilitate bacterial TMA and subsequently host TMAO production (db/db choline 4w group, n = 10) or 0.5% choline in drinking water and 250 mg/kg metformin (db/db choline + metformin 4w group, n = 10). Db/Lean mice (n = 10) were used as a control. Faecal samples were collected after 4 weeks of choline administration. At the end of the treatment these 40 mice received bolus dose of choline (100 mg/kg) to evaluate the overall capacity to produce TMAO and the ability of metformin to decrease TMAO production after acute substrate load. For this, plasma samples were collected immediately before and 2 h after choline load (100 mg/kg). Plasma samples were collected from tail veins during the dark cycle; samples were centrifuged, and the plasma was stored at -80 °C for future analysis. All experiments were performed in a blinded manner.

### Glucose, lactate and insulin concentrations

The plasma insulin concentration was determined using a Sensitive Insulin RIA Kit (EMD Millipore, Billerica, MA, USA). Blood glucose was measured using a MediSense Optium glucometer from Abbott Diabetes Care (Maidenhead, UK). Lactate in plasma was determined using a kit from Instrumentation Laboratory (Lexington, MA, USA).

### Determination of choline, TMAO and TMA concentrations

Determination of TMAO concentration in blood plasma was performed by ultra-performance liquid chromatography-tandem mass spectrometry (UPLC/MS/MS) in positive ion electrospray mode, as described previously^42^. Choline quantification and trimethylamine concentration determination were performed as described previously^19^.

### Microbial cultures, TMA production, assay of choline uptake and FMO3 activity

To test microbial TMA production, two bacterial species were used: *Klebsiella pneumoniae* (obtained from the Microbial Strain Collection of Latvia (MSCL), strain number 535) and *Proteus mirabilis (*MSCL, strain number 590). Bacteria were maintained on LB agar plates. A single colony was used as inoculum for each experiment. To test the effect of metformin on choline-dependent TMA production, bacteria were grown in M9 mineral broth supplemented with 0.2% casamino acids (Merck, Darmstadt, Germany)^43^ with 27 mM choline as the carbon source, with or without 27 mM metformin addition. Metformin concentration of 27 mM was chosen as it represents levels of a drug that could be achieved in the intestines after administration of high (≥ 850 mg) metformin doses to patients^14,38^.

Micro-anaerobic cultivations were performed essentially as described previously^19^. Briefly, to ensure anaerobic conditions 2 ml test tubes were filled with broth and covered with airtight caps; the test tubes were kept still in an incubator at + 37 °C. Samples were harvested after 4 and 8 h of cultivation, fixed with formic acid to 5% final concentration and centrifuged. Supernatants were frozen (−80 °C) and stored until further analyses.

The effect of metformin on bacterial growth was assessed by spectrophotometric recording of *K. pneumoniae* and *P. mirabilis* growth dynamics in anaerobic tubes, as previously described^44^. Briefly, microbial cells were grown in M9 broth with glucose as the sole carbon source overnight. Cells were harvested by centrifugation, washed and resuspended in fresh M9 broth with different carbon sources, glucose or choline (final concentration of 27 mM) with or without metformin (final concentration 27 mM). Airtight, clear glass HPLC bottles (vol. 4.5 mL, diameter 1 cm) were top-filled with different microbial suspensions and left to incubate at + 37 °C. The absorbance of the microbial suspensions was recorded using a WPA colorimeter (Biochrom Ltd, UK) set to 590 nm. Choline uptake in *K. pneumoniae* cells was measured using [^3^H]-choline as a substrate as described previously^19^.

FMO3 activity was determined in C57Bl6 lean mice liver microsomal fraction based on methimazole/DTNB assay as described previously^45^. Choline-TMA-lyase enzymatic activity was determined based on a method previously described by Roberts and colleagues^46^ with modifications as indicated below. *P. mirabilis* bacterial cells were lysed, and the reaction was carried out in an inflatable glove bag filled with nitrogen gas to ensure anaerobic conditions. An enzymatic activity assay was performed using non-labelled choline, and the conversion of choline to TMA was determined with UPLC/MS/MS. The reaction mixture contained clarified bacterial lysate in lysis buffer and the necessary co-factors (1 mM SAM, 10 mM NaDT, and 2 mM NADH (Sigma-Aldrich, Schnelldorf, Germany)). Metformin was added to the reaction mixture (27 mM final concentration) to evaluate its effect on choline-TMA-lyase activity. The mixture was then allowed to incubate for 15 min before the initiation of the reaction by adding choline (1 mM final concentration). Baseline samples were collected, and the reaction was carried out for 2 h at room temperature in the dark. Afterwards, samples were harvested, fixed with formic acid (1.5% final concentration) and stored at -80 °C until further analysis.

### Choline-TMA-lyase gene expression

To determine if a metformin–induced decrease in TMA production was associated with changes in the transcription of choline metabolism-related genes in gut bacteria, we tested the transcription of three *K. pneumoniae* choline-TMA-lyase complex coding genes (cmcA, CutC, CutD)^23^. Gene expression was evaluated in *K. pneumoniae* after 4 and 8 h of cultivation with and without 27 mM metformin. Total RNA was isolated and purified from *K. pneumoniae* cell pellets (stored at -80 °C) using a PureLink RNA extraction kit (ThermoFisher Scientific, USA) as recommended by the manufacturer's protocol. First-strand cDNA synthesis and quantitative RT-PCR analysis for genes were performed as described previously^47^. Relative expression levels for each gene were calculated with the ∆∆Ct method and normalized to the expression of the glyceraldehyde 3-phosphate dehydrogenase gene (GAPDH) gene. The primer sequences used for the quantitative RT-PCR analysis were as follows: 5′- TGTTGATGTCGTTGTGCGGA-3′ (Fw cmcA), 5′- CGTCGAGCTTATCGGCTATGA-3′ (Rv cmcA), 5′-TCGGTAACCAGACCCGTAAA-3′ (Fw cutC), 5′-GGCGCGAGTTTTCTCTTCTA-′3 (Rv cutC), 5′-GATTAACACCGCCGTCGAAA-3′ (Fw cutD), 5′-TCCACCAGCCATTCGAGATT-3′ (Rv cutD), 5′- ACCGTTCGTCTGGAAAAAGC-3′ (Fw GAPDH) and 5′- ACGAAGTTGTCGTTCAGTGC-3′ (Rv GAPDH).

### DNA isolation from faecal samples and qPCR analysis

Total DNA from mice faeces was isolated^48^ using FastDNA SPIN Kit for Soil (MP Biomedicals) following the manufacturer’s instructions. qPCR analysis and relative bacteria quantification was performed by using KAPA SYBR FAST master mix (Sigma-Aldrich) and MIC qPCR Cycler (Bio-Molecular Systems) and using the following conditions: Polymerase activation 95 °C for 3 min; Touchdown 10 cycles [95 °C for 15 s, 65 °C for 15 s-0.5 C per cycle decrease, 68 °C for 10 s]; Cycling 60 cycles [95 °C for 5 s, 60 °C for 15 s, 72 °C for 15 s]. Primers specific for selected bacterial species and genera were selected from literature and checked using the Primer-BLAST tool^49^ and are listed in Supplementary Table 1. The relative bacteria composition was determined for each genera or species by using ΔΔCt method and normalized to the Ct of universal bacteria primer (TotBact‑176).

### Statistical methods

Data are presented as the mean ± SD (standard deviation) for bacterial data and as the mean ± SEM (standard error of the mean) for animal data. Statistically significant differences in the mean values were evaluated based on data normality analysis using a one-way ANOVA or Kruskal–Wallis test. A t-test was used when only two groups were compared, repeated measures t-test was used when changes over time were compared within one group. If ANOVA or Kruskal–Wallis test provided *P* < 0.05, Tukey’s or Dunn’s test was performed, respectively, and the differences were considered significant when *P* < 0.05. None of the animal samples were excluded from the analysis. After performing ROUT analysis to identify outliers several samples were excluded from analysis for bacterial composition data in faecal samples and exclusions are indicated in the respective figure legends. The data were analysed using GraphPad Prism statistical software (GraphPad Inc., San Diego, CA, USA).

### Ethics approval

The experimental procedures involving animals were performed in accordance with the guidelines of the European Community (Directive 2010/63/EU) and local laws and policies, and all of the procedures were approved by the Latvian Animal Protection Ethical Committee, Food and Veterinary Service, Riga, Latvia. Studies involving animals are reported in accordance with the ARRIVE guidelines.

## Supplementary information

Supplementary information.

## References

[1] Karlsson FH (2013). Gut metagenome in European women with normal, impaired and diabetic glucose control. Nature.

[2] Qin J (2012). A metagenome-wide association study of gut microbiota in type 2 diabetes. Nature.

[3] Tang WH (2013). Intestinal microbial metabolism of phosphatidylcholine and cardiovascular risk. N. Engl. J. Med..

[4] Wang Z (2011). Gut flora metabolism of phosphatidylcholine promotes cardiovascular disease. Nature.

[5] Dambrova M (2016). Diabetes is associated with higher trimethylamine N-oxide plasma levels. Exp. Clin. Endocrinol Diabetes.

[6] Lever M (2014). Betaine and trimethylamine-N-oxide as predictors of cardiovascular outcomes show different patterns in diabetes mellitus: an observational study. PLoS ONE.

[7] Miao J (2015). Flavin-containing monooxygenase 3 as a potential player in diabetes-associated atherosclerosis. Nat. Commun..

[8] Chen S (2019). Trimethylamine N-oxide binds and activates PERK to promote metabolic dysfunction. Cell Metab..

[9] Flory JH, Keating SJ, Siscovick D, Mushlin AI (2018). Identifying prevalence and risk factors for metformin non-persistence: a retrospective cohort study using an electronic health record. BMJ Open.

[10] Forslund K (2015). Disentangling type 2 diabetes and metformin treatment signatures in the human gut microbiota. Nature.

[11] Wu H (2017). Metformin alters the gut microbiome of individuals with treatment-naive type 2 diabetes, contributing to the therapeutic effects of the drug. Nat. Med..

[12] Lee H (2018). Modulation of the gut microbiota by metformin improves metabolic profiles in aged obese mice. Gut Microbes.

[13] Bauer PV (2018). Metformin alters upper small intestinal microbiota that impact a glucose-SGLT1-sensing glucoregulatory pathway. Cell Metab..

[14] Proctor WR, Bourdet DL, Thakker DR (2008). Mechanisms underlying saturable intestinal absorption of metformin. Drug Metab. Dispos.

[15] Han Y (2019). Effect of metformin on all-cause and cardiovascular mortality in patients with coronary artery diseases: a systematic review and an updated meta-analysis. Cardiovasc. Diabetol..

[16] Luo F (2019). Metformin in patients with and without diabetes: a paradigm shift in cardiovascular disease management. Cardiovasc. Diabetol..

[17] Koeth RA (2013). Intestinal microbiota metabolism of L-carnitine, a nutrient in red meat, promotes atherosclerosis. Nat. Med..

[18] Koeth RA (2014). gamma-Butyrobetaine is a proatherogenic intermediate in gut microbial metabolism of L-carnitine to TMAO. Cell Metab..

[19] Kuka J (2014). Suppression of intestinal microbiota-dependent production of pro-atherogenic trimethylamine N-oxide by shifting L-carnitine microbial degradation. Life Sci..

[20] Wu WK (2019). Identification of TMAO-producer phenotype and host-diet-gut dysbiosis by carnitine challenge test in human and germ-free mice. Gut.

[21] Martinez-del Campo A (2015). Characterization and detection of a widely distributed gene cluster that predicts anaerobic choline utilization by human gut bacteria. mBio.

[22] Craciun S, Marks JA, Balskus EP (2014). Characterization of choline trimethylamine-lyase expands the chemistry of glycyl radical enzymes. ACS Chem. Biol..

[23] Kalnins G (2015). Structure and function of CutC choline lyase from human microbiota bacterium *Klebsiella pneumoniae*. J. Biol. Chem..

[24] Madiraju AK (2014). Metformin suppresses gluconeogenesis by inhibiting mitochondrial glycerophosphate dehydrogenase. Nature.

[25] McCreight LJ, Bailey CJ, Pearson ER (2016). Metformin and the gastrointestinal tract. Diabetologia.

[26] Stynen B (2018). Changes of cell biochemical states are revealed in protein homomeric complex dynamics. Cell.

[27] Al-Obaide MAI (2017). Gut microbiota-dependent trimethylamine-N-oxide and serum biomarkers in patients with T2DM and advanced CKD. J. Clin. Med..

[28] Chou RH (2019). Trimethylamine N-oxide, circulating endothelial progenitor cells, and endothelial function in patients with stable angina. Sci. Rep..

[29] Li T, Chen Y, Gua C, Li X (2017). Elevated circulating trimethylamine N-oxide levels contribute to endothelial dysfunction in aged rats through vascular inflammation and oxidative stress. Front Physiol..

[30] Nafisa A (2018). Endothelial function and dysfunction: impact of metformin. Pharmacol. Ther..

[31] Sardu C (2019). Effects of metformin therapy on coronary endothelial dysfunction in patients with prediabetes with stable angina and nonobstructive coronary artery stenosis: the CODYCE multicenter prospective study. Diabetes Care.

[32] Eskens BJ, Zuurbier CJ, van Haare J, Vink H, van Teeffelen JW (2013). Effects of two weeks of metformin treatment on whole-body glycocalyx barrier properties in db/db mice. Cardiovasc. Diabetol..

[33] Hamidi Shishavan M (2017). Metformin improves endothelial function and reduces blood pressure in diabetic spontaneously hypertensive rats independent from glycemia control: comparison to vildagliptin. Sci. Rep..

[34] Beli E, Prabakaran S, Krishnan P, Evans-Molina C, Grant MB (2019). Loss of diurnal oscillatory rhythms in gut microbiota correlates with changes in circulating metabolites in type 2 diabetic db/db mice. Nutrients.

[35] Huo T (2009). Metabonomic study of biochemical changes in the serum of type 2 diabetes mellitus patients after the treatment of metformin hydrochloride. J. Pharm. Biomed. Anal..

[36] Velebova K (2016). The effect of metformin on serum levels of Trimethylamine-N-oxide in patients with type 2 diabetes/prediabetes and chronic heart failure. Diabetologia.

[37] Latkovskis G (2018). Loop diuretics decrease the renal elimination rate and increase the plasma levels of trimethylamine-N-oxide. Br. J. Clin. Pharmacol..

[38] Bailey CJ, Wilcock C, Scarpello JH (2008). Metformin and the intestine. Diabetologia.

[39] Herring TI, Harris TN, Chowdhury C, Mohanty SK, Bobik TA (2018). A Bacterial microcompartment is used for choline fermentation by *Escherichia coli* 536. J. Bacteriol..

[40] Kilkenny C (2010). Animal research: reporting in vivo experiments: the ARRIVE guidelines. Br. J. Pharmacol..

[41] McGrath JC, Drummond GB, McLachlan EM, Kilkenny C, Wainwright CL (2010). Guidelines for reporting experiments involving animals: the ARRIVE guidelines. Br. J. Pharmacol..

[42] Dambrova M (2013). Meldonium decreases the diet-increased plasma levels of trimethylamine N-oxide, a metabolite associated with atherosclerosis. J. Clin. Pharmacol..

[43] Sack JS (2008). Structural basis for the high-affinity binding of pyrrolotriazine inhibitors of p38 MAP kinase. Acta Crystallogr. D Biol. Crystallogr..

[44] Seim H, Löster H, Claus R, Kleber H-P, Strack E (1982). Formation of γ-butyrobetaine and trimethylamine from quaternary ammonium compounds structure-related to l-carnitine and choline by *Proteus vulgaris*. FEMS Microbiol. Lett..

[45] Rose, R. L. Measurements of flavin-containing monooxygenase (FMO) activities. *Curr Protoc Toxicol* **Chapter 4**, Unit4 9, 10.1002/0471140856.tx0409s13 (2002). 10.1002/0471140856.tx0409s13 20945301

[46] Roberts AB (2018). Development of a gut microbe-targeted nonlethal therapeutic to inhibit thrombosis potential. Nat. Med..

[47] Liepinsh E (2016). Decreased acylcarnitine content improves insulin sensitivity in experimental mice models of insulin resistance. Pharmacol. Res..

[48] Ferrand J (2014). Comparison of seven methods for extraction of bacterial DNA from fecal and cecal samples of mice. J. Microbiol. Methods.

[49] Ye J (2012). Primer-BLAST: a tool to design target-specific primers for polymerase chain reaction. BMC Bioinform..

