# Supplementary material for: Metformin decreases bacterial trimethylamine production and trimethylamine N-oxide levels in db/db mice
Source: Sci Rep. 2020 Sep 3;10:14555. doi: 10.1038/s41598-020-71470-4 (PMC7471276; doi:10.1038/s41598-020-71470-4)
Supplement: Supplementary file 1 — Supplementary information. [file 41598_2020_71470_MOESM1_ESM.docx]

**Metformin decreases bacterial trimethylamine production and trimethylamine N-oxide levels in db/db mice**

Kuka Janis^a,*^, Videja Melita^a,b^, Makrecka-Kuka Marina^a^, Liepins Janis^c^, Grinberga Solveiga^a^, Sevostjanovs Eduards^a^, Vilks Karlis^a^, Liepinsh Edgars^a^, Dambrova Maija^a,b^

^a^ Latvian Institute of Organic Synthesis, Aizkraukles Str. 21, Riga, LV-1006, Latvia

^b^ Rīga Stradiņš University, Faculty of Pharmacy, Dzirciema Str. 16, Riga, LV-1007, Latvia

^c^ University of Latvia, Institute of Microbiology and Biotechnology, Jelgavas Str. 1, Riga LV-1004, Latvia

*Corresponding author

[janis.kuka@farm.osi.lv](mailto:janis.kuka@farm.osi.lv)

**Supplementary results**

**Supplementary figure 1.** Effects of choline and metformin administration on relative presence of bacteria in gut microbiota from db/db mice. The results are mean of 8 or 10 animals. One sample was excluded in db/Lean group, one in db/db control group and two samples were excluded in choline + metformin group for Desulfovibrio species after identified as outliers by ROUT analysis. * P<0.05 vs. db/db control group, ^$^ P<0.05 vs. db/db choline group (Kruskal-Wallis test followed by Dunn’s multiple comparison test).

**Supplementary figure 2.** Effects of metformin on FMO3 activity in liver tissues of C57Bl6 lean mouse. Results are mean of 5 replicates.

**Supplementary Figure 3.** Effects of metformin (27 mM) on choline consumption under anaerobic conditions. The results are the mean ± SD of 3 independent replicates for *K. pneumoniae* and 4 replicates for *P. mirabilis*. * P<0.05 vs. respective time point control (Student`s t-test).

**Supplementary methods**

**DNA isolation and qPCR analysis**

Total DNA from mice faeces was isolated using FastDNA™ SPIN Kit for Soil (MP Biomedicals) following the manufacturer’s instructions. qPCR analysis and relative bacteria quantification was performed by using KAPA SYBR® FAST master mix (Sigma-Aldrich) and MIC qPCR Cycler (Bio-Molecular Systems) and using the following conditions: Polymerase activation 95°C for 3 min; Touchdown 10 cycles [95°C for 15 seconds, 65°C for 15 seconds-0.5 C per cycle decrease, 68°C for 10 seconds]; Cycling 60 cycles [95°C for 5 seconds, 60°C for 15 seconds, 72°C for 15 seconds]. Primers specific for selected bacterial species and genera were selected from literature and checked using the Primer-BLAST tool [1] and are listed in Supplementary Table 1. The relative bacteria composition was determined for each genera or species by using ΔΔCt method and normalized to the Ct of universal bacteria primer (TotBact 176).

**Supplementary Table 1** Primer sequences used in this study

| **Primer name** | **Specificity** | **Forward primer sequence** (5'->3') | **Reverse primer sequence (5'->3')** | **Amplicon length, b** | **Reference** |
| --- | --- | --- | --- | --- | --- |
| TotBact-176 | All bacteria | TCCTACGGGAGGCAGCAG | TATTACCGCGGCTGCTGG | 176 | [2] |
| Desulfovibrio-136 | Desulfovibrio genus | CCGTAGATATCTGGAGGAACATCAG | ACATCTAGCATCCATCGTTTACAGC | 136 | [3] |
| P.Mirab.-67 | Proteus mirabilis | TGTGGCGGGTACTAATGCAA | CGCCTCTAACATGCGGTACA | 67 | [4] |
| Olsenella-95 | Olsenella genus | CTTACCAGGGCTTGACATCTTGG | ACGACACGAGCTGACGACAG | 95 | [5] |

**References**

1. Ye J, Coulouris G, Zaretskaya I, Cutcutache I, Rozen S, Madden TL. Primer-BLAST: a tool to design target-specific primers for polymerase chain reaction. BMC Bioinformatics. BioMed Central; 2012;13:134.

2. Fuks G, Elgart M, Amir A, Zeisel A, Turnbaugh PJ, Soen Y, et al. Combining 16S rRNA gene variable regions enables high-resolution microbial community profiling. Microbiome [Internet]. BioMed Central Ltd.; 2018 [cited 2020 Jul 1];6:17. Available from: https://microbiomejournal.biomedcentral.com/articles/10.1186/s40168-017-0396-x

3. Fite A, Macfarlane GT, Cummings JH, Hopkins MJ, Kong SC, Furrie E, et al. Identification and quantitation of mucosal and faecal desulfovibrios using real time polymerase chain reaction. Gut. BMJ Publishing Group; 2004;53:523–9.

4. Fukumoto H, Sato Y, Hasegawa H, Saeki H, Katano H. Development of a new real-time PCR system for simultaneous detection of bacteria and fungi in pathological samples. Int J Clin Exp Pathol. E-Century Publishing Corporation; 2015;8:15479–88.

5. Li X, Jensen BB, Højberg O, Noel SJ, Canibe N. Development of a species-specific TaqMan-MGB real-time PCR assay to quantify Olsenella scatoligenes in pigs offered a chicory root-based diet. AMB Express. Springer Verlag; 2018;8:1–13.
